# The role of rilpivirine in Southern Africa

**DOI:** 10.4102/sajhivmed.v20i1.825

**Published:** 2019-05-29

**Authors:** Michelle A. Moorhouse, Karen Cohen

**Affiliations:** 1 Wits Reproductive Health and HIV Institute, University of the Witwatersrand, Johannesburg, South Africa; 2 Division of Clinical Pharmacology, Department of Medicine, University of Cape Town, Cape Town, South Africa

**Keywords:** HIV, Rilpivirine, Antiretrovirals, Non-nucleoside reverse transcriptase inhibitor, Southern Africa

## Abstract

Rilpivirine, a second-generation non-nucleoside reverse transcriptase inhibitor (NNRTI), is included as an option in first-line antiretroviral therapy (ART) for antiretroviral-naïve individuals in treatment guidelines in high-income countries, including the United States and many European countries. Rilpivirine is available in a single-tablet fixed-dose combination, has a favourable tolerability profile and is of relatively low cost. However, rilpivirine has reduced efficacy in patients commencing ART at high viral loads. Therefore, baseline viral load testing is required before commencing rilpivirine, and it is not recommended for patients commencing therapy with a viral load greater than 100 000 copies/mL. Rilpivirine is not included in the treatment regimens recommended by the World Health Organization (WHO), which form the basis of treatment guidelines in many lower- and middle-income countries. Some patients commencing standard first-line regimens experience treatment-limiting toxicity. A low-cost rilpivirine-containing fixed-dose combination would potentially be a useful addition to treatment options available in South Africa and other countries in the region, for patients who do not tolerate standard first-line ART. In this article, we explore the utility of rilpivirine as an option in ART in South Africa and the region in the context of current public-sector regimens. We consider what role rilpivirine might play if first-line therapy moves to a dolutegravir-based regimen, as has already happened in some lower- and middle-income countries, including Botswana, Kenya and Brazil. Finally, we describe emerging evidence for rilpivirine in the prevention of HIV transmission.

## Introduction

Rilpivirine, a second-generation non-nucleoside reverse transcriptase inhibitor (NNRTI), is currently approved for the treatment of HIV-1 infection in more than 80 countries around the world. It is included as an option in first-line antiretroviral therapy (ART) for antiretroviral-naïve individuals in treatment guidelines in high-income countries, including the United States and many European countries.^1,2^ Rilpivirine is available in a single-tablet fixed-dose combination, has a favourable tolerability profile and is relatively low cost.^3^ Because of reduced efficacy in patients commencing ART at high viral loads, baseline viral load testing is required before commencing rilpivirine, and it is not recommended for patients commencing therapy with a viral load greater than 100 000 copies/mL. Rilpivirine has not been included in the treatment regimens recommended by the World Health Organization (WHO), which form the basis of treatment guidelines in many lower- and middle-income countries. South Africa has the largest antiretroviral programme in the world, and purchases antiretrovirals in quantities similar to both the President’s Emergency Program for AIDS Relief (PEPFAR) and the Global Fund. A low-cost, well-tolerated, single-tablet regimen would potentially be a useful addition to treatment options available in South Africa and other countries in the region.

The suitability of any antiretroviral agent for use within large-scale treatment programmes is determined by a number of factors. These include efficacy; tolerability profile, preferably with no need for laboratory monitoring for safety issues; interactions with commonly prescribed drugs, especially those used to treat coinfections such as tuberculosis and hepatitis viruses; convenience and availability of fixed-dose combinations; availability of paediatric formulations; safety in pregnancy; and affordability.

In this article, we explore the utility of rilpivirine as an option in ART in South Africa and the region, in the context of current public-sector regimens. We consider what role rilpivirine might play if first-line therapy moves to a dolutegravir-based regimen, as has already happened in some lower- and middle-income countries, including Botswana, Kenya and Brazil. Finally, we describe emerging evidence for rilpivirine in the prevention of HIV transmission.

## Rilpivirine for the management of HIV infection: Efficacy and tolerability

The efficacy and tolerability of rilpivirine compared to efavirenz in antiretroviral-naïve individuals was explored in a phase 2b dose-finding study,^4,5^ in the phase 3 ECHO^6^ and THRIVE^7^ studies (data from these two registrational studies were pooled for analysis at weeks 48 and 96)^8,9,10,11^ and the single-tablet regimen (STaR) study.^12^ In all of these studies, with the exception of the phase 2b dose-finding study, rilpivirine was associated with fewer adverse events leading to treatment discontinuation than efavirenz (including neuropsychiatric events, rash and dyslipidaemia).

The phase 2b study explored three different doses of rilpivirine compared to efavirenz 600 mg. The rilpivirine doses explored were 25 mg, 75 mg and 150 mg, and the study was powered to detect a dose response across the arms at a 5% significance level.^5^ The study was extended to follow participants out to 192 weeks from 96 weeks and found similar viral suppression and immunological efficacy across the rilpivirine and efavirenz arms, and the rilpivirine 25 mg dose was taken forward into phase 3.

The ECHO and THRIVE studies demonstrated non-inferior efficacy of rilpivirine compared to efavirenz overall. However, in these studies, which compared rilpivirine 25 mg with efavirenz 600 mg, both in combination with two nucleoside reverse transcriptase inhibitors (NRTIs), rates of virological failure and emergence of viral resistance were higher in individuals with high baseline viral loads (> 100 000 copies/mL) receiving rilpivirine than efavirenz at both 48^13^ and 96 weeks.^10^ In the ECHO and THRIVE studies, 23/686 (3%) of participants taking rilpivirine discontinued because of an adverse event, versus 52/682 (8%) taking efavirenz.^13^ In a systematic review of randomised clinical trials, comparing safety and neuropsychiatric adverse events of efavirenz with other agents, participants had double the risk of discontinuation because of adverse drug reactions with efavirenz-containing ART regimens than with rilpivirine-containing regimens (RR: 2.0, 95% confidence interval [CI]: 1.0 to 3.8; RD: 4.1, 95% CI: 1.3 to 6.8).^14^ Side effects of rilpivirine were uncommon; the two most common side effects were headache in 2% and insomnia in 2% of participants.^15^

In the STaR study, both rilpivirine and efavirenz were administered in combination with tenofovir disoproxil fumarate (TDF) and emtricitabine (FTC) as single-tablet regimens. STaR stratified participants at randomisation according to their baseline viral load (≤ *or* > 100 000 copies/mL) and demonstrated superiority of the rilpivirine arm at weeks 48 and 96 overall at viral loads below 100 000 copies/mL (for higher viral loads, the rilpivirine arm was non-inferior).^12,15^

The switching at low HIV-1 RNA into fixed-dose combinations (SALIF) study demonstrated non-inferior efficacy of rilpivirine (RPV)/TDF/FTC in maintaining virological suppression at 48 weeks compared to efavirenz (EFV)/TDF/FTC (both administered as a single tablet regimen [STR]) in people living with HIV who were virologically suppressed on a NNRTI-based regimen prior to randomisation. In the SALIF study, there were more discontinuations and treatment-limiting adverse events in the rilpivirine arm. This most likely reflects the effect of being switched to a new rilpivirine-containing regimen, as opposed to continuing on an efavirenz-based regimen (randomisation ensured a balance of participants who were previously on nevirapine regimen across both arms, approximately 45%).^16^

Observational data from the Swiss HIV cohort followed up patients who were initiated on or switched to the RPV/TDF/FTC STR for two years. The main reasons for switches were for regimen simplification or efavirenz-related neuropsychiatric adverse events. The study population were mainly antiretroviral-experienced and virologically suppressed. At 24 months, 96% of treatment-experienced and 100% of treatment-naïve patients remained virologically suppressed. Among those who were switched because of neuropsychiatric adverse events, 78.3% experienced improvement at 12 months.^17^ These results were similar to those of other observational cohorts assessing switches to the STR.^18,19,20^

In summary, rilpivirine has lower efficacy than efavirenz in patients with high viral loads at ART initiation. This limits the usefulness of rilpivirine-based regimens in first-line ART in resource-constrained settings. Most large programmes in lower- and middle-income countries do not routinely quantify viral load at baseline, and adding this test in large programmes would add considerable treatment costs. However, switch studies of virologically suppressed patients have shown good efficacy of rilpivirine in maintaining suppression.^16,17,18,19,20,21^ Rilpivirine may therefore be a useful option for patients needing to switch for tolerability issues, especially those relating to neuropsychiatric adverse events.

## Rilpivirine for the management of HIV infection: Other important considerations for programmatic settings

### Drug interactions

Rilpivirine is mainly metabolised by cytochrome P450 3A4, rendering it vulnerable to the effects of drugs that are cytochrome P450 3A4 inhibitors or inducers. Its solubility and therefore absorption are pH dependent, requiring a low pH. Because of this, rilpivirine cannot be co-administered with proton pump inhibitors, and when prescribed with histamine-2 receptor antagonists and antacids, doses need to be separated to allow rilpivirine absorption.^22^

Rilpivirine cannot be administered with rifampicin-containing regimens, which are still the mainstay of tuberculosis management in many lower- and middle-income countries where the burden of tuberculosis is the highest. Rifampicin may reduce the rilpivirine concentrations substantially,^23^ putting patients at risk of virological failure. There is a similar interaction with rifabutin.^22^ This means that all patients on rilpivirine who develop rifamycin-sensitive tuberculosis would need to be switched to alternative ART. This adds to programmatic complexity, as well posing a risk to patients who may be switched to a less well-tolerated regimen, potentially risking virological failure. However, this is not a problem unique to rilpivirine, as many ARVs require either a treatment change (such as atazanavir) or a change in dose (such as ritonavir-boosted lopinavir and dolutegravir) when co-administered with rifampicin. With the move to ‘test and treat’ resulting in earlier ART initiation at higher CD4+ counts, and with increased rollout of various TB prevention therapies, this may become less of an issue as incident TB rates are likely to decline.

### Convenience

Fixed-dose combinations are preferred in large programmes, not only for their benefits in terms of adherence but also for the benefits they offer in terms of simplicity of supply chain, distribution, stock management, storage and prescription. Internationally, both rilpivirine and efavirenz are available co-formulated with either TDF and FTC, or tenofovir alafenamide (TAF) and FTC. TAF is a prodrug of tenofovir, which is potentially associated with less bone and renal toxicity than TDF because of lower plasma tenofovir exposure.^24,25^ Unfortunately, none of the rilpivirine fixed-dose combinations, or formulations of either drug containing TAF, are currently available in South Africa.

### Paediatric formulations

Currently, there are no specific paediatric formulations of rilpivirine available and the adult formulation can only be used in children from 12 years and weighing 35 kg or more. Where possible, within large-scale programmes, harmonisation of adult and paediatric regimens is preferred. This is not currently possible with rilpivirine.

### Safety in pregnancy

Pregnancy can affect rilpivirine concentrations by its effect on cardiac output, protein binding, volume of distribution and cytochrome P450 3A4 activity.^26^ Rilpivirine concentrations are reduced during pregnancy, especially during the third trimester,^26,27^ as is seen with many other antiretrovirals. In two studies that investigated the pharmacokinetic characteristics of rilpivirine in pregnant women, nearly all the women had rilpivirine concentrations above the IC_90_ (the concentration of drug required to inhibit viral replication by 90%). This suggests that no dose adjustment is required despite the reduced rilpivirine exposures during pregnancy. In both studies, the women maintained virological suppression and the infants were not HIV-infected.^26,27^

The Antiretroviral Pregnancy Registry (APR) still has relatively low numbers of rilpivirine-exposed pregnancies. For first trimester exposures to rilpivirine, there was one birth defect in 202 pregnancies (0.5%; 95% CI: 0.0%, 2.7%); for second trimester exposures, there were three birth defects in 247 pregnancies (1.2%; 95% CI: 0.3%, 3.5%).^28^ No specific teratogenic concerns have emerged to date.

### Affordability

In South Africa, rilpivirine currently has a ‘single exit’ price of around ZAR 50.00 (around US $3.70) for a 30-day supply versus ZAR 175.00 (around US $13.00) for efavirenz.^29^ Efavirenz is unlikely to decrease in price any further and is therefore unlikely to achieve price parity with rilpivirine. This does not include any costs related to managing side effects related to the use of efavirenz, which is associated with higher adverse event-related discontinuations compared to rilpivirine across several studies.^14^

## Role of rilpivirine in programmes using efavirenz-based first-line therapy

Internationally, treatment guidelines have moved to recommending ART initiation irrespective of WHO clinical stage or CD4+ count.^30^ Benefits of ART initiation are modest for those with earlier stage disease, and need to be weighed up against the potential harms, which include side effects and toxicity from ART when initiated in asymptomatic patients at high viral loads.

A systematic review including 42 randomised controlled trials found that the relative risk for discontinuations because of adverse events was higher for efavirenz compared to most other first-line options, including low-dose efavirenz (400 mg), rilpivirine, TDF, atazanavir and maraviroc, and that neuropsychiatric adverse events were common with efavirenz.^14^ Notably, most of the studies included were conducted in predominantly white populations. Black Africans have a much higher prevalence of efavirenz slow metaboliser genotypes than white people (17% vs. 3% in South Africa),^31^ which may result in more frequent efavirenz-related neuropsychiatric adverse events.

Efavirenz is superior to rilpivirine for programmatic use in a standard first-line ART regimen because it has higher virological efficacy at high viral loads, is available in fixed-dose combination formulations, can be prescribed with rifampicin and is safe in pregnancy. Rilpivirine cannot be prescribed with rifamycin-containing TB treatment. Although rilpivirine is cheap, it has reduced efficacy at high baseline viral loads and cannot therefore be used for first-line therapy without pre-ART initiation viral loads. Pre-ART viral loads are not routinely performed in southern African ART programmes and would considerably increase total cost to the healthcare system of the first-line regimen. In addition, data on rilpivirine use in pregnancy are limited and the reduction in rilpivirine exposure during the third trimester of pregnancy is concerning.^26,27,32^

However, for patients with a contraindication to efavirenz (e.g. history of psychosis) or who experience severe neuropsychiatric side effects on efavirenz, alternatives are currently limited. Nevirapine cannot be prescribed for women initiating ART with CD4+ counts above 250 cells/µL or men initiating ART with CD4+ counts above 400 cells/µL because of the risk of severe hepatotoxicity. In addition, nevirapine is associated with a higher frequency of severe adverse events, particularly treatment discontinuations, than efavirenz.^8^ The only other alternative in current guidelines for patients with high CD4+ counts is to initiate ritonavir-boosted lopinavir. As ritonavir-boosted lopinavir is used in second-line therapy, this limits future treatment options. As rilpivirine has better tolerability than efavirenz,^14^ a rilpivirine-based regimen would be a very useful alternative for patients with contraindications to efavirenz or treatment-limiting toxicity once initiated on efavirenz-based ART.

## Role of rilpivirine as programmes move to dolutegravir-containing first-line therapy

Dolutegravir is newly included in current WHO guidelines as a recommendation for first-line ART and is starting to be rolled out across several lower- and middle-income countries. Dolutegravir has demonstrated robustness with a formidable barrier to resistance, good tolerability and superiority to both efavirenz and ritonavir-boosted lopinavir in clinical trials, in both antiretroviral-naïve and experienced patients.^33,34^ In these studies, dolutegravir’s tolerability is likely to have contributed to the superior efficacy demonstrated. The main dolutegravir-related treatment-emergent adverse event reported in these studies was insomnia.^33,34^ Dolutegravir was found to be protective of discontinuations because of adverse events in a network meta-analysis that included 34 032 patients on first-line ART.^35^ Dolutegravir was also shown to have superior efficacy to both efavirenz and rilpivirine in the network meta-analysis.^35^

Because of the relatively small number of carefully selected participants included in registrational studies of newer antiretrovirals, more information regarding the safety profile may emerge only after registration, once the use of the antiretroviral becomes more widespread. Since dolutegravir’s approval, observational cohorts have reported neuropsychiatric adverse events, including depression, anxiety and suicidal ideation in patients receiving dolutegravir.^36,37,38,39^ This has been seen with other integrase inhibitors and has been postulated as a possible class effect.^40,41,42,43^ More recently, data have suggested^44^ that dolutegravir may also be associated with weight gain in a number of cohorts as well as clinical studies. Potentially, rilpivirine could be used in people living with HIV who experience intolerance to dolutegravir as the transition to dolutegravir-based first-line ART rolls out.

Furthermore, a prospective study was set up in Botswana to quantify the incidence of neural tube defects with efavirenz exposure. This cohort also considers other birth outcomes and includes a number of patients on dolutegravir. It recently showed a worrying signal of increased incidence of neural tube defects with periconception use of dolutegravir.^45^ If confirmed, this signal will raise a dilemma regarding the use of dolutegravir in women at risk of falling pregnant.

## Dolutegravir in combination with rilpivirine: A nucleoside-sparing treatment option

Recent data from the SWORD 1 and 2 studies of dolutegravir in combination with rilpivirine suggest that this dual therapy regimen may be effective maintenance regimens in virologically suppressed patients.^46^ The SWORD studies randomised virologically suppressed participants with no history of virological failure to continue their current ART regimen or switch to dolutegravir and rilpivirine. Switching to the dual therapy regimen was found to be non-inferior to continuing the initial regimen over 48 weeks. This is despite the inherent bias of the switch study design against the drug being studied, where switching may result in adverse events that could result in virological failure because of non-adherence. In addition, observational data from several cohorts have demonstrated the efficacy of this regimen in achieving and maintaining virological suppression in patients with varying degrees of antiretroviral experience.^47,48,49^ A fixed-dose combination of rilpivirine and dolutegravir was recently approved by the Food and Drug Administration (FDA),^50^ based on the results of the SWORD studies. However, the inability to co-administer with rifamycin-containing TB treatment limits the usefulness of this coformulation in lower- and middle-income settings with high TB burdens.

## Rilpivirine in long-acting injectable antiretroviral regimens

The combination of long-acting rilpivirine and the integrase inhibitor cabotegravir is being studied in an injectable therapy for the treatment and prevention of HIV-1 infection. The phase 2b LATTE 2 study randomised patients to oral cabotegravir plus abacavir plus lamivudine and long-acting cabotegravir plus rilpivirine injected 4 weekly, and found that the injectable dual therapy regimen was well tolerated and had efficacy similar to the oral regimen.^51^ Further studies, such as first long-acting injectable regimen (FLAIR) and ART as long-acting suppression (ATLAS), are currently underway to evaluate this dual therapy injectable regimen. However, there is still significant research needed before this regimen could be considered for programmatic use in lower- and middle-income countries. This would need to include data on TB co-infected and pregnant patients.

## The role of rilpivirine in HIV prevention: Pre-exposure prophylaxis

Currently the only approved and WHO-recommended pre-exposure prophylaxis (PrEP) option is oral tenofovir-based PrEP,^30^ which has demonstrated its efficacy in preventing HIV infection in multiple randomised controlled trials^52^ and demonstration projects in different target populations, with more evidence continuing to accumulate. However, there are those for whom oral tenofovir-based PrEP is not an appropriate option, either on account of contraindications to the drug itself, or on account of other factors related to oral formulation or the requirement to take pills regularly. For such individuals, alternative PrEP options are essential.

Early studies of long-acting rilpivirine (alone or in combination with a long-acting integrase inhibitor, cabotegravir) confirmed the initial safety, acceptability and pharmacokinetic characteristics of the formulation.^53,54^ Plasma, rectal and cervical fluid sampling and limited rectal and vaginal biopsies after single doses of 300 mg – 1200 mg long-acting rilpivirine in one of these studies demonstrated prolonged rilpivirine exposure in plasma as well as the genital tract for 84 days. In this study, one female participant subsequently experienced incident HIV-1 (wild-type virus) infection after a single episode of unprotected vaginal intercourse approximately 41 days after she received the 300 mg rilpivirine injection. Viraemia peaked on day 115, and at this point, a mixed population of 101 K/E was detected and ART was initiated. Resistance was selected by high levels of viral replication in the face of low rilpivirine concentrations. The viral population reverted to predominantly wild-type by day 199. This incident infection illustrates the risk of subtherapeutic concentrations of long-acting agents that are not high enough to prevent infection but are sufficient to select for resistance.^56^

A subsequent phase 1 study enrolled 36 HIV-negative participants and alternately assigned them to receive one intramuscular dose of long-acting rilpivirine, either 1200 mg or 600 mg. The safety and acceptability findings were in line with previous studies. These findings were confirmed in a multiple dose phase of this study.^57^

HPTN 076 is a double-blind, randomised study, which compared the safety of long-acting rilpivirine1200 mg every 8 weeks to placebo, following a 4-week run-in oral phase, for PrEP in low-risk, sexually active HIV-uninfected women. The study demonstrated no significant differences between the two arms with respect to adverse events. It also showed that for 92% of participants, plasma rilpivirine concentrations were above the protein-adjusted IC_90_. Currently, HPTN 076 study has only reported safety, acceptability and pharmacokinetic findings,^58,59^ and the long-acting rilpivirine will not be taken forward into PrEP efficacy studies at this stage.

To date, no studies using rilpivirine oral formulations as PrEP have been conducted, except where it has been used as an initial run-in phase to establish tolerability prior to progressing onto the administration of injectable formulations.

## The role of rilpivirine in HIV prevention: Post-exposure prophylaxis

While a number of PrEP randomised controlled trials have been conducted with various drug regimens, there are no randomised studies on antiretroviral regimens for post-exposure prophylaxis (PEP). Most data supporting the use of PEP are derived from animal transmission models, prevention of mother-to-child transmission trials, observational studies of healthcare workers receiving PEP following occupational HIV exposure, and observational cohorts and case studies of PEP following potential sexual exposure.

Adherence to the full 28-day course of PEP has been shown to be poor, with only 56.6% (95% CI 50.9–62.2%; *t*^2^ 0.25) of those eligible completing the course,^60^ and lower in adolescents than in adults. A significant proportion of PEP discontinuation is related to adverse events, indicating that better tolerated PEP regimens are needed. A systematic review of observational data concluded that tenofovir-based PEP regimens were better tolerated than those using zidovudine, while the optimal third drug to use was a little less clear. While raltegravir was well tolerated, other factors such as twice daily dosing, availability and cost were identified as limitations to the use of raltegravir as the third drug, particularly in resource-limited settings.^61^

Subsequently, a number of PEP observational studies using newer antiretrovirals have been undertaken. One single-arm study and one observational cohort used rilpivirine for PEP. The single arm study used a STR of rilpivirine combined with TDF and FTC for 28 days in 100 HIV-uninfected men who have sex with men. Post-exposure prophylaxis completion rates were high at 92% and adherence rates at 98.6% (standard deviation [s.d.], 2.4) by pill count. Discontinuations for adverse events were low (1%).^62^ These results are similar to the findings of a French observational cohort study including 129 participants, which evaluated the safety, tolerability, adherence and efficacy of a 28-day course of single-tablet RPV/TDF/FTC commenced within 48 hours of potential exposure to HIV.^63^

In these studies, adverse events were commonly reported by participants but were of low grade, and there were no seroconversions. With the high number of participants completing their PEP regimens in these studies, rilpivirine is an attractive option as a third drug for PEP regimens, offering the advantages of cost and coformulation (although the coformulation is not available in many countries). With more lower- and middle-income countries introducing dolutegravir into first-line ART, another potential advantage of rilpivirine-based PEP is the lack of overlapping resistance profiles between rilpivirine and dolutegravir.

## Ethical consideration

This article followed all ethical standards for a research without direct contact with human or animal subjects.

## Conclusion

Many lower- and middle income countries use efavirenz-based regimens for first-line ART. Efavirenz has good efficacy and relatively good tolerability, is available in fixed-dose combination STR at low cost, is safe in pregnancy and can be used concomitantly with rifampicin-containing TB treatment. However efavirenz causes treatment-limiting toxicity in some individuals and should be used with caution in psychiatric disease.

Rilpivirine cannot replace efavirenz in standard first-line therapy. Rilpivirine is not optimal for patients with high baseline viral loads because of reduced efficacy, and cannot be prescribed with rifampicin. In addition, there is a paucity of rilpivirine safety data in pregnancy, and the decrease in rilpivirine concentrations during pregnancy is concerning: more data in pregnancy are required to inform recommendations on use in pregnancy. However, rilpivirine is generally well tolerated and may be a useful alternative for people living with HIV who cannot tolerate efavirenz or in whom it is contraindicated. If rilpivirine is initiated in treatment-naïve patients, a baseline viral load is mandatory because of reduced efficacy at high viral loads in phase 3 trials, and if the baseline viral load is more than 100 000 copies/mL, an alternative drug is preferable.

With increasing use of dolutegravir as more country programmes transition to dolutegravir-based first-line ART, emergent neuropsychiatric adverse events are being reported, suggesting an overlapping toxicity profile with current efavirenz-based regimens. For patients who experience neuropsychiatric or other toxicity or with contraindications to current or future first-line ART regimens, rilpivirine may be a useful alternative.
